# Analysis of Mechanical Properties and Printing Orientation Influence of Composite Resin for 3D Printing Compared to Conventional Resin

**DOI:** 10.3390/ma17225626

**Published:** 2024-11-18

**Authors:** Leonardo V. Araújo, Fabiana S. Figuerêdo de Siqueira, Rayssa F. Cavaleiro de Macedo, Felipe S. Gomes, Gustavo G. Castro, Daniela B. Dibai, Etevaldo M. Maia Filho, Rudys R. J. Tavarez

**Affiliations:** 1 Post Graduated Program in Dentistry Program, Ceuma University, São Luís 65075-120, MA, Brazilfabiana.siqueira@ceuma.br (F.S.F.d.S.); danielabassifisio@gmail.com (D.B.D.); emmaiafilho@yahoo.com.br (E.M.M.F.); 2 Post Graduated Program in Dentistry Program, Federal Maranhao University, São Luís 65085-582, MA, Brazil; rayssafcm@gmail.com (R.F.C.d.M.); ofelipegomesfsg@gmail.com (F.S.G.)

**Keywords:** 3D printing, composite resin, flexural strength, microhardness, surface roughness

## Abstract

This study aimed to compare the flexural strength, surface roughness, and microhardness of a resin for three-dimensional (3D) printing and a conventional composite resin and to evaluate whether the printing orientation influences these properties. To evaluate the flexural resistance, test specimens were produced and divided into four groups: three groups of resins for 3D printing with inclinations of 0°, 45°, and 90° and one group of conventional composite resin. Forty discs were produced and subjected to a sandpaper-polishing sequence, and the surface roughness was measured using a roughness meter. The Vickers microhardness (HV) test was performed at three different points, and the average was obtained. The results were subjected to ANOVA statistical analysis and Tukey’s test. There were statistical differences in the flexural strength and microhardness between the conventional resin and the resin used for 3D printing. No statistical difference in surface roughness was observed. The resin for 3D printing exhibited lower flexural strength and microhardness than conventional composite resins. We concluded that the resin for 3D printing had lower flexural strength and HV than the conventional composite resin but similar surface roughness. The printing orientation did not affect the flexural strength, whereas the hardness increased as the printing angle increased.

## 1. Introduction

Computer-aided design and manufacturing (CAD/CAM) was introduced in dentistry in the 1980s, revolutionizing the production of prosthetic parts both in laboratories and offices, substantially reducing the number of steps carried out to create an indirect restoration and, consequently, the time to complete a restorative treatment [1]. The CAD/CAM technology is divided into two phases: CAD, which consists of the design of the restoration carried out using computer programs, and CAM, which represents the production stage of the restoration designed in the CAD phase. The CAM phase can be performed using two methods: subtractive manufacturing (MS) or milling and additive manufacturing (MA) or three-dimensional (3D) printing [2].

Subtractive manufacturing involves creating objects by milling or machining blocks and/or solid discs using milling cutters. It is widely used in materials such as zirconia (ZrO_2_), polymethylmethacrylate (PMMA), and glass ceramics, and its main advantages include a reduction in operational costs and human error compared to manual production [3]. However, its disadvantages include high consumption of cutters, wastage of raw materials, and difficulty in producing complex geometries [3,4].

Three-dimensional printing transforms a virtual object (CAD file) into a physical object by superimposing thin layers of raw material, which is a common characteristic of all 3D printing technologies [5]. Developed in the 1980s and patented in 1986 by Chuck Hull with the creation of a production technology called stereolithography (SLA), 3D printing began to be widely used both commercially and industrially after the patents expired, enabling the development of new technologies such as Digital Light Processing (DLP) and Mask Stereolithography (MSLA) [2,5,6].

Stereolithography technology uses a UV laser with a 200–500 nm wavelength that covers the entire printing area and polymerizes the layers of photosensitive polymers. The DLP and MSLA technologies use the radiation produced by a UV light projector and light-emitting diodes to solidify the polymer according to the contour of the object to be produced, making the printing process faster with lower investment and input costs compared with SLA [6,7].

Three-dimensional printing is used in several specialties such as oral and maxillofacial surgery, implant dentistry, endodontics, and periodontics to produce surgical guides and models for surgical planning [8], orthodontics and dental prosthetics for the production of models, complete dentures, tray individuals [9], provisional indirect restorations [10], and more recently, for definitive or long-term indirect restorations [11]. To perform 3D printing of any device, it is necessary to use software to position and orient the object in relation to the ground, which can assume a parallel or perpendicular orientation or with variations in angulation. This orientation is directly related to the overlapping of material layers for the formation of the object during 3D printing, which can interfere with its mechanical properties [12].

With the increasing application of 3D printing, new polymeric materials are being developed to produce prostheses and restorations that can remain in the mouth for long periods [7]. Such materials have a composition similar to conventional resins and are categorized as nano-hybrid composite resins, presenting an organic matrix such as bisphenol A glycidyl methacrylate (Bis-GMA), Urethane dimethacrylate (UDMA), Bisphenol A/ethylene glycol/methyl dimethacrylate (Bis-EMA), and triethylene glycol dimethacrylate (TEGDMA) as well as inorganic ceramic fillers such as zirconia and silanized silica and barium glass, aiming to increase their mechanical and biocompatibility properties [7,13].

Three-dimensional printing plays a revolutionary role in dentistry, making it possible to obtain restorations that can be used temporarily or even long-term. Thus, evaluating the mechanical properties of the materials used to obtain these restorations and how impression orientation could influence these properties is essential for determining the biomechanical characteristics in comparison with restorations obtained using conventional techniques.

Considering this scenario, the objective of this study is to compare the flexural strength, surface roughness, and microhardness of a nano-hybrid composite resin with silanized ceramic and zirconia fillers for 3D printing and a conventional nano-hybrid composite resin and evaluate whether printing orientation influences these properties. The null hypotheses tested were as follows: (1) there is no difference in the mechanical properties between the materials tested, and (2) the printing orientation does not influence the mechanical properties of the resin for 3D printing.

## 2. Materials and Methods

### 2.1. Experimental Design

The materials used were the nano-hybrid composite resin with silanized ceramic and zirconia fillers priZma 3D Biocrown (Makertech Labs, Tatuí, São Paulo, Brazil) for 3D printing and the conventional nano-hybrid composite resin FORMA A1B (Ultradent Brasil, Indaiatuba, São Paulo, Brazil) (Table 1).

The control group consisted of specimens from the conventional nano-hybrid composite resin (n = 10), and the specimens from the resin for 3D printing were divided into three subgroups: (1) 0°BC0 (n = 10), (2) 45°BC45 (n = 10), and (3) 90°BC90 (n = 10), according to the printing orientation (0°, 45°, and 90°) in relation to the ground, totaling four groups, three of which were made of resin for 3D printing and one of a conventional composite resin.

### 2.2. Preparation of Test Specimens

The resin from the resin group for 3D printing was used to print the specimen in conjunction with a Photon D2 3D printer (Anycubic, Shenzhen, Guangdong, China) with DLP technology after mechanized shaking for 40 min to achieve homogenization, according to the manufacturer’s instructions.

**Figure 1:**
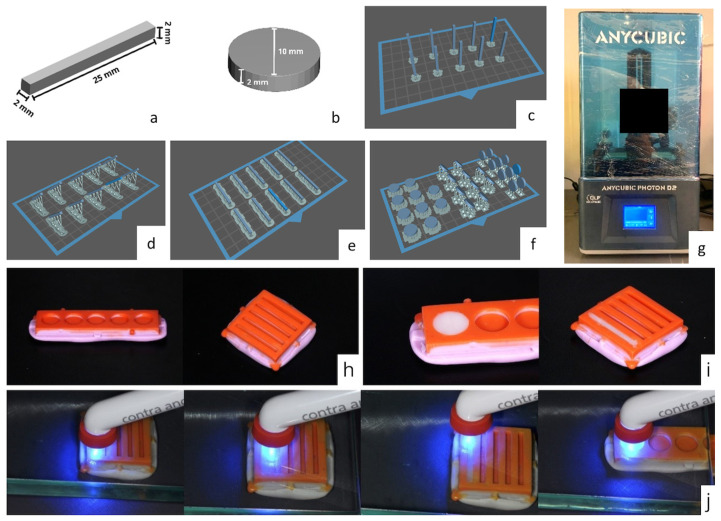
Design of the specimens in CAD software and import into the software for 3D printing: (**a**) bar, (**b**) disc, (**c**) slash 90°, (**d**) bar 45°, (**e**) bar 0°, (**f**) disc at 0°, 45°, and 90°. (**g**) Anycubic Photon D2 printer. Silicone molds: (**h**) molds for bar and disc. (**i**) Conventional composite resin condensed in the molds. (**j**) Photoactivation of specimens in conventional composite resin.

The specimens were printed with a layer thickness of 50 µm, and the printing time parameters were defined based on a previously printed calibration object so that the specimen presented the specified dimensions (Figure 1).

After printing, the specimen was subjected to post-processing in two stages: washing in isopropyl alcohol for 10 min [6] to remove excess unpolymerized resin in a Form Wash washer (Formlabs Inc., Somerville, MA, USA) and post-curing in a Form Cure (Formlabs Inc., Somerville, MA, USA), a UV light cabinet with a wavelength of 405 nm, for 60 min, according to the manufacturer’s guidelines. The supports were removed, and the surface and the region with the support were regularized using a diamond disc and abrasive rubbers (Dhpro, Paranaguá, Paraná, Brazil) at low speed with the aid of a bench motor and straight handpiece (Beltec, Araraquara, São Paulo, Brazil).

All specimens were measured using digital calipers (Mitutoyo Corp., Kanagawa, Japan) to validate their length, width, thickness, and diameter.

Specimens from the conventional composite resin group were fabricated in condensation silicone molds obtained from a matrix designed using the CAD program Meshmixer version 3.5.474 (Autodesk, San Francisco, CA, USA) and printed. The molds followed the dimensions specified in the ISO 4049:2019 standard [14]. The composite resin was condensed with a metal spatula in a single increment until it filled the mold space, the excess was removed, and a 10 mm glass plate was placed on the mold to homogenize the surface. Photopolymerization of the specimens was performed using a single calibrated operator. The bar-shaped specimens (n = 10) were photopolymerized in three areas (specimen divided into three thirds) for 60 s in each area on the glass plate, whereas in the disc-shaped specimens (n = 10), photopolymerization was carried out at a central point on the glass plate using a Radii Plus photopolymerizer (SDI Limited, Bayswater, Victoria, Australia) [15] (Figure 1).

### 2.3. Flexural Strength Test (σ)

The specimens used to evaluate the flexural resistance of the resin group for 3D printing (n = 30) were designed using a CAD program Meshmixer versin 3.5.474 (Autodesk, San Francisco, CA, USA), in the shape of a bar with dimensions of L × W × E of 25 (±2) × 2 (±0.1) × 2 (±0.1) mm, following the recommendations of the International Organization for Standardization ISO 4049:2019 for testing polymer-based restorative materials [14].

The bar-shaped specimen was subjected to the 3-point flexural strength test on an Instron 3342 universal testing machine (Instron, Norwood, MA, USA) with a load cell of 500 N and a speed of 1 mm/min until rupture, and values expressed in N were converted into flexural strength (σ) in Mpa using the following formula:

σ = 3FL/2bd^2^,

where

σ = flexural strength
F = load (force) at the fracture point
L = length of support span
b = width of the sample
d = sample thickness

### 2.4. Surface Roughness Test (Ra)

Disc-shaped specimens with a diameter of 10 mm and thickness of 2 mm were designed (n = 30) to evaluate the surface roughness and microhardness [16]. The bar and disk format designs were exported in STL (standard tessellation language) format and imported into the Chitubox Basic program version 1.9.5 (Chitubox, Shenzhen, Guangdong, China), a specific software for 3D printing.

The disc-shaped specimens were positioned in templates designed and printed with rigid resin, and the face with the supports was polished with water sandpaper with grits #600, #1200, #1500, and #2000 on an automated polishing machine (Ecomet 250, Buehler, Lake Bluff, IL, USA) for 30 s at 50 revolutions per minute (rpm) and a vertical pressure of 10 N on the specimens. After polishing, the discs were numbered on the template [17].

An SJ-301 rugosimeter (Mitutoyo Corp., Kanagawa, Japan) with ISO 21920-2:2021 [18] measurement parameters was used, and three measurements were performed in different directions. The considered value was the arithmetic mean (Ra) of the highest peak and the deepest point.

### 2.5. Microhardness Test

After the surface roughness test, the specimens were positioned in an HMG-V microhardness meter (Shimadzu do Brasil, Barueri, São Paulo, Brazil) to perform the Vickers microhardness (HV) test using a pyramidal-shaped diamond indenter with a load of 50 g for 30 s in three different areas and measured at 20× magnification to obtain an average value [19].

#### Statistical Analysis

The Shapiro–Wilk test was conducted, and we found that in one of the groups, the flexural strength and surface roughness were not normally distributed. After data normalization using the Box–Cox transformation, analysis of variance (ANOVA) and Tukey’s post hoc test were used to test the hypothesis that the 3D printing orientation did not influence the flexural strength, surface roughness, and HV of the resins [20].

The effects of 3D printing orientation on the flexural strength, surface roughness, and HV were calculated using Partial Eta squared (partial ƞ^2^).

All tests were performed using the SPSS 26.0 statistical program (IBM, Armonk, NY, USA). The significance level established was 5%.

## 3. Results

**Figure 2:**
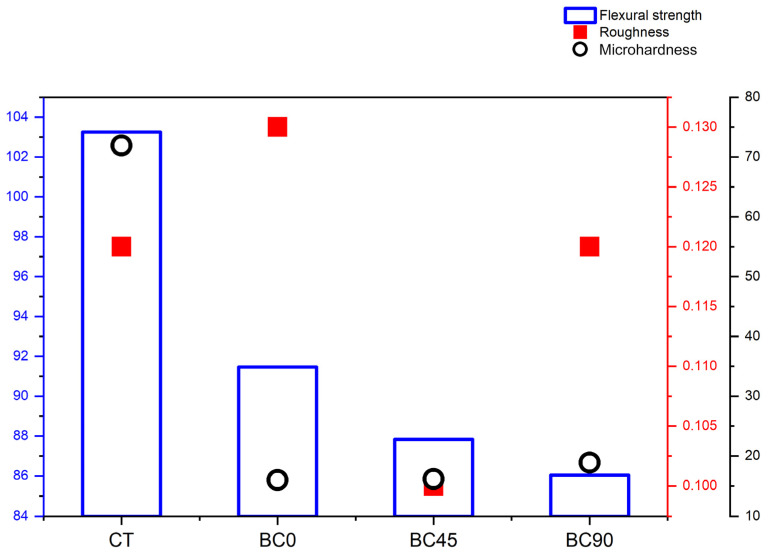
Multi-axis graph showing average values of flexural strength, roughness, and microhardness according to 3D printing orientation.

Figure 2 shows a multi-axis graph of the average values (standard deviations) of the flexural strength, surface roughness, and HV according to the 3D printing orientation.

There was a decrease in the flexural resistance values of the 3D printing groups with an increase in the printing angle; however, this was not statistically significant (*p* > 0.05). The flexural resistance of the composite resin group was significantly greater than that of the 3D printing group (*p* < 0.001).

There was no statistically significant difference in the surface roughness between the resin in the composite group and the groups with resin for 3D printing (*p* = 0.387).

The average HV of the resin in the composite resin group was significantly higher than that of the resin in the 3D printing resin group (*p* < 0.001). The BC90 group exhibited a higher average HV than the BC0 and BC45 groups. Table 2 shows the mean values (standard deviations) and 95% confidence interval (95% CI) of the groups.

This section is divided into several subsections. A concise and precise description of the experimental results, their interpretation, and experimental conclusions that can be drawn.

## 4. Discussion

The results of this study showed that the first Ho proposal, which indicated no difference in the mechanical properties between 3D printed resin and conventional composite resin, was partially accepted. The flexural strength and HV properties showed significant differences between the two materials; however, no significant difference was observed in surface roughness. The second Ho proposal, which indicates that the printing orientation did not influence the mechanical properties of the materials, was also partially accepted. The flexural strength and microhardness variables were not influenced by the printing orientation, whereas the surface roughness variable was.

The resin for 3D printing showed lower flexural resistance on the three slopes than the conventional composite resin. The values obtained were also lower than the minimum resistance required for restorative materials based on Type 1, Class 2, and Group 2 polymers (100 Mpa for the occlusal face), according to ISO 4049:2019 [14]. Several studies that evaluated the flexural strength of impression resins indicated for temporary restorations found values between 49.87 and 159.9 Mpa, which would fit the resin tested in this category, diverging from the manufacturer’s indication [15,17,21,22,23,24].

The literature shows that some factors can influence the mechanical properties of printed resins, such as the post-curing time associated with the increase in temperature during the process [22,25]. A duration of 30 min at 60 °C significantly increased the flexural strength of the resin for permanent restorations, as measured by biaxial tests [25,26]. In this study, the post-printing stage followed the manufacturer’s guidelines, which indicated a post-curing time of 30–60 min without increasing the temperature.

Hardness is a crucial characteristic of restorative materials, and HV tests indicate a high density of these materials, making them capable of resisting wear processes in the oral cavity [3]. The tested resin showed statistically significantly lower microhardness values than the conventional resin group. Studies have shown that printing resins containing inorganic fillers tend to present higher HV values than materials based on PMMA [22,27].

Bora et al. characterized the composition of several resins for 3D printing and compared their mechanical properties with those of conventional resins and found that a greater amount of inorganic filler in the resins resulted in higher HV values [3]. These results corroborate the results of this study, as the resin in the conventional group had 67% inorganic filler by weight, whereas the resin in the 3D printing group had less than 35%.

The resin for 3D printing has UDMA as its main organic constituent, a high-molecular-weight and high-viscosity monomer, and diluent monomers that promote increased fluidity of the resin, enabling the 3D printing process to take place without failure [28]. Lin et al. studied the mechanical properties of different organic matrices used in the composition of printing resins and showed that UDMA in greater proportions increased the flexural strength and degree of conversion but reduced the microhardness [29]. These findings are consistent with those of the present study.

The inorganic phase of the tested resin was composed of silanized zirconia mixed oxide and other filler particles such as silanized silica and barium glass. The incorporation of spherical silica particles reduced the surface roughness while maintaining the resistance characteristics [30]. The presence of barium glass improves the optical properties of the resin, increases the radiopacity and translucency, and decreases the microhardness, which reduces the wear generated on the antagonists [30]. The addition of zirconia increases microhardness, increasing the resin’s resistance to wear [3].

The surface roughness Ra was used in this study due to its simplicity and ability to provide a representative indication of surface roughness is an effective parameter for analyzing roughness in composite resins and other dental materials [31]. In this study, the tested resins did not show significant differences in the Ra test, which means that the resins obtained by 3D printing allowed finishing and polishing equivalent to those of conventional composite resins [17]. The surface roughness standard for composite resins after polishing is 0.2 µm, and values above this standard increase the risk of bacterial plaque accumulation [31]. Surface roughness patterns below this value were obtained for the resin tested at three printing inclinations, corroborating values reported in the literature [17].

Printing orientation is a critical aspect in obtaining satisfactory results in 3D printing and involves determining the direction in which the restoration is built in relation to the horizontal plane, which directly affects the number of layers and time required for the final print [32]. The influence of this parameter on the mechanical properties of the resins was previously reported [33]. The reduction in flexural resistance values in the tested resin as the printing orientation angle increased has been reported in the literature, indicating that impressions parallel to the horizontal plane, with layer construction perpendicular to the direction of forces, tend to offer greater resistance [16,21,32]. However, resins for different applications such as surgical guides and occlusal plates used in printers with technologies that differ from those used in the present study may present divergent results [33,34].

Other studies evaluated the surface roughness and microhardness of specimens printed in different orientations, showing roughness levels above the minimum standard for resins, which is justified by the formation of small union lines between overlapping layers of the material [16,35]. However, these studies did not consider mechanical polishing of the surfaces after printing, which differs from the methodology and results of the present study. The literature also shows that changing the printing orientation does not change the microhardness values, because this property is more strongly related to the filler content present in the resins [19].

Another aspect to be considered is the printing accuracy between the technologies, owing to the dimensional reproduction capacity and precision of the object. The literature reports the superiority of DLP printers over MSLA [36,37]. Chen et al. found that the flexural strength of resins for restorations printed using a DLP printer was higher; however, this difference could be reduced by increasing the post-curing time [22]. The decrease in the costs of the DLP equipment and the positive results obtained in previous research support the choice of this technology in the present study [38].

Mechanical properties play a fundamental role in restorative materials as they must resist functional and parafunctional occlusal forces while maintaining their anatomical and surface polishing characteristics. Conventional composite resins are widely used in the manufacture of direct and indirect restorations because of their flexural resistance and surface roughness, which allow them to remain in the mouth for long periods [39,40,41]. However, new resin materials for 3D printing have been developed with improved inorganic filler contents, which provide mechanical properties closer to those of conventional resins while maintaining the benefits of low cost and increased production speed [13].

The present study has strengths, including the use of stable and precise printing technology, as well as the comparison between materials with similar characteristics and clinical applications, which differ in their composition and energy source for conversion; however, further studies are necessary to characterize new resins for 3D printing, to identify the types and percentages of inorganic fillers present in these materials, as these constituents can contribute to improving flexural resistance and hardness characteristics. Some limitations of the study need to be considered, such as the absence of thermocycling, which could influence the mechanical properties of the tested material, and the lack of evaluation of different post-curing times.

The results of this study can be useful to dental surgeons in the selection and indication of materials for 3D printing, and the tested material can be indicated with caution as a long-lasting temporary material, especially for people with limited economic resources where ceramic materials cannot be used due to their higher cost.

Furthermore, studies that simulate the conditions of the oral cavity in vivo and new materials with improvements in the inclusion of filler particles or polymerization techniques still need to be tested to evaluate the longevity and maintenance of the characteristics of these materials. In addition, other parameters could complement surface roughness such as root mean square surface roughness (Rq), and maximum height of the profile (Rz). There are also other methods in the literature to evaluate the shape and texture of the surface, such as profilometry and atomic force electron microscopy.

## 5. Conclusions

Based on the results obtained, it can be concluded that, although flexural strength decreases with increasing printing angle in the 3D printing groups, this variation is not statistically significant. The flexural strength and microhardness of the composite resin group are significantly higher than those of the 3D printing groups. Surface roughness is similar between the composite resin and the 3D printing resin. Studies that simulate oral cavity conditions in vivo are essential for evaluating the longevity and maintenance of the characteristics of these new materials.

## Figures and Tables

**Table 1:** Materials used, composition, and batch.

Material	Composition	Batch
**Forma Resin (Ultradent from Brazil)**	Bis-GMA; Bis-EMA; TEGDMA; BHT; PEGDMA; UDMA; Ytterbium trifluoride; fillers based on silane-treated ceramics, silanized silica, silanized silica-zirconium oxide, and barium glass.	D0IEU
Prizma 3D Biocrown (Makertech Labs)	UDMA > 40%; other methacrylated monomers > 20%; TPO < 3%; Mixed Silanized Zirconia Oxide < 10%; other filler particles (silanized silicas and barium nano- and microglass) < 25%; Pigments < 2%; blockers, stabilizers, and coactivators < 2%.	209623

Bis-GMA: bisphenol A glycidyl methacrylate; BIS-EMA: Bisphenol A/ethylene glycol/methyl dimethacrylate; TEGDMA: Triethylene glycol dimethacrylate; BHT: butyl hydroxytoluene; PEGDMA: Polyethylene glycol dimethacrylate; UDMA: Urethane dimethacrylate; TPO: Trimethylbenzoyl-diphenyl phosphine oxide.

**Table 2:** Mean values (standard deviation) and 95% confidence interval (95% CI) of the groups evaluated.

		Mean (Standard Deviation)	95% CI		
			Lower Bound	Upper Bound	ƞ^2^ Parcial
Flexural strength (Mpa)	CT	103.26 (13.84) ^A^	93.35	113.16	0.386
	BC0	91.46 (7.83) ^B^	85.86	97.06	
	BC45	87.83 (5.56) ^B^	83.85	91.81	
	BC90	86.03 (5.89) ^B^	81.81	90.24	
Surface roughness (µm)	CT	0.12 (0.02) ^A^	0.10	0.13	
	BC0	0.13 (0.04) ^A^	0.10	0.17	
	BC45	0.10 (0.02) ^A^	0.09	0.12	
	BC90	0.12 (0.05) ^A^	0.08	0.16	
Microhardness (HV)	CT	71.93 (2.51) ^A^	70.13	73.73	
	BC0	15.97 (1.68) ^B^	14.77	17.18	0.995
	BC45	16.15 (1.55) ^B^	15.04	17.26	
	BC90	18.89 (1.51) ^C^	17.80	19.98	

Different capital letters vertically statistically significant difference (*p* < 0.05).

## Data Availability

The raw data supporting the conclusions of this article will be made available by the authors on request.
